# The spectrum of idiopathic inflammatory myopathies in Western Australia: epidemiological characteristics and mortality over time

**DOI:** 10.1007/s00296-023-05475-3

**Published:** 2023-10-11

**Authors:** Johannes Nossent, Helen Keen, David B. Preen, Charles A. Inderjeeth

**Affiliations:** 1 Department of Rheumatology, Sir Charles Gairdner Hospital, Perth, Australia; 2 Rheumatology Group, School of Medicine, University of Western Australia, 35 Stirling Highway (M503), Perth, WA Australia; 3 Department of Rheumatology, Fiona Stanley Hospital, Perth, Australia; 4 School of Population and Global Health, University Western Australia, Perth, Australia

**Keywords:** Idiopathic inflammatory myopathies, Incidence rate, Crude mortality rate, Survival, Causes of death

## Abstract

To determine long term overall and subgroup specific incidence rates and associated mortality for idiopathic inflammatory myopathies (IIM) in a population wide study. We included patients hospitalised between 1980 and 2015 with incident IIM as defined by relevant diagnostic codes for dermatomyositis (DM) polymyositis (PM), inclusion body myositis (IBM), other IIM and overlap myositis (OM) in the Western Australia Health Hospital Morbidity Data Collection (*n* = 846). Trends over time for annual incidence rate per million population (AIR) were analysed by least square regression and Kaplan–Meier survival and mortality rates (MR)/100 person years compared with a matched control group (*n* = 3681). The averaged AIR for all IIM was 19 (CI 10.4–27.5) and stable over time with point prevalence reaching 205.3 (CI 185.6–226.6) per million in 2015. Over time, the AIR for DM 5.0 (CI 0.6–9.4) and IBM 3.3 (CI 0.7–9.6) was stable, while AIR decreased for PM (*p* < 0.01) and increased for other IIM (*p* < 0.01) and OM (*p* < 0.01). IBM patients were eldest at diagnosis (68 years, CI 59–77) with male preponderance in IBM (53.4%) and other IIM (55.8%) groups. Crude mortality (54.5 vs 41.3%), MR ratio (6.65 vs 5.91) and 5 (65.8% vs 71.6%) and 10-year (52.5% vs 58.7%) survival were all worse for IIM patients (all *p* < 0.05). IBM patients had highest MR (10.1; CI 8.38–12.14) and lowest 10-year survival (39.2%). While cardiovascular disease and cancer were predominant causes of death, they were proportionally lower in IIM patients, where respiratory and rheumatic disease were more frequent causes of death. While the overall incidence of IIM in WA was stable over 35 years, the spectrum of IIM has changed significantly with increases especially in other IIM and OM. The overall prognosis with IIM remains guarded with 10-year survival just over 50%.

**Supplementary Information:**

The online version contains supplementary material available at 10.1007/s00296-023-05475-3.

## Introduction

Idiopathic inflammatory myopathies (IIMs) are a group of chronic acquired immune-mediated diseases often classified into subgroups, such as dermatomyositis (DM), polymyositis (PM) and inclusion body myositis (IBM) The pathogenesis of IIM remains poorly understood and differs between subgroups with vasculopathy a key feature of DM and protein misfolding and dysfunction seen in IBM [1–3]. Epidemiologic data support a role for infections, preceding lung disease, physical exertion, collagen implants, ultraviolet radiation, cancer and smoking in developing IIM phenotypes [4, 5]. Also, an expanding number of autoantibodies have been recognised in IIM patients to assist with subgroup classification, but there is no clear consensus on their pathogenic or prognostic value [6]. Australian data on IIM are relatively scarce with an overall IIM incidence of 7–8 /million for biopsy confirmed cases in South Australia and Victoria and an annual incidence of IBM of 14.5/million in Western Australia (WA) [7–12]. Long-term outcome studies in IIM have revealed an increased standardised mortality rate in addition to increased risks for cardiovascular disease and cancer [13–15]. We performed a long-term population wide study of the overall epidemiological characteristics and mortality in WA for patients hospitalised with IIM and analysed these features and trends in distinct subgroups.

## Methodology

This was a population-level observational cohort study including all persons over 15 years of age residing in WA between 1 January 1980 and 31 December 2014 for which data were derived from the Western Australia Rheumatic Disease Epidemiological Registry (WARDER). WARDER contains routinely collected validated health data from all public and private health care organisations for the state of Western Australia for around 250,000 patients with inflammatory rheumatic diseases over the period 1980–2015. WARDER data are sourced from four different datasets (WA Health Hospital Morbidity Data Collection) (HMDC), Emergency Department Data Collection (EDDC), WA Cancer Registry and WA Death Registry. WARDER also contains data from a control group of age and gender matched hospitalised patients, which were free of inflammatory rheumatic disease during the study period. Patient data in WARDER were effectively linked through a validated process of probabilistic matching and clerical review to provide de-identified longitudinal health data for each included individual based on the clinical diagnoses made by physicians and then translated to ICD codes [16, 17]. The WARDER database has been successfully applied to the clinical-epidemiological study of rheumatic diseases [18–20]. We included in this study patients with a IIM diagnosis recorded according to International Classification of Diseases (ICD-9CM/ICD-10-AM) in hospital discharge codes derived from Australian Classification Index pathways and coding rules (https://www.ihacpa.gov.au/resources/clinical-coding-practice-framework). ICD discharge coding has been found to have a high positive predictive value for DM, PM and IBM case finding in separate studies [11, 21–23], while for this study IIM was defined according to the recently validated algorithm by Hannah et al. [24] that includes subgroups paraneoplastic PM/DM (M36.0), DM (M33.9, M33.1, M33.0) PM (M33.2), inflammatory myopathy (G72.4), other myositis (M60.8) and myositis unspecified (M60.9) when accompanied by ILD codes (J84.1, J84.9, J99.1). This coding algorithm has a positive predictive value of 89% against a probable diagnosis of IIM as per clinical expert opinion. Also, for this study we added on patients with overlap myositis (OMM) (G73.7) in line with recommendations [25]. With the support of the WA Clinical Coding Authority, backward mapping to relevant ICD9-CM codes was performed for the period 1980–1999 (see Suppl Table 1). Finally, each IIM patient identified was matched to five non-exposed hospitalised controls on gender, Indigenous status, year and age at IIM diagnosis. The final dataset for this study contained sociodemographic data, length and type of admission (e.g. intensive care), all principal and secondary diagnoses (up to 20) for all earlier and subsequent hospital contacts as well as details from cancer and death notices for each participant. The Western Australia Department of Health Ethics Committee provided approval for this project (project no. 2016.24, approval date September 1, 2016, extension granted September, 2021).

### Statistical analyses

Primary outcomes were average annual incidence rates (AIR) and point prevalence per 31.12.2014, both expressed per million population (as registered for WA by the Australian Bureau of Statistics) and mortality rates (MR) per 100 person years with 1998 data used as middle population for overall AIR estimations. Descriptive statistics include median and interquartile range (IQR) for continuous variables compared by non-parametric methods (Kruskal–Wallis), categorical data described with a frequency and proportion and group comparisons tested with odds ratios (OR) and Fisher’s exact test. Incidence rates were calculated per 1000 person years with 95%CI derived from Poisson distribution, and changes in rates over time were assessed by linear least squares regression analysis using the coefficient of determination (R-squared, *R*^2^) as the goodness-of-fit measure where higher coefficients (range 0–1) indicate a better fit for increasing or decreasing incidence rates over time. Survival data were based on Kaplan–Meier estimates with logrank testing for subgroup comparisons and Cox regression analysis to determine hazard ratios (HR with 95%CI) for death for specified risk factors. All statistical analyses were performed using SPSS software v28.0 (IBM, USA) and OpenEpi software with two-sided *p*-values (*p*) < 0.05 considered to be statistically significant.

## Results

**Fig. 1:**
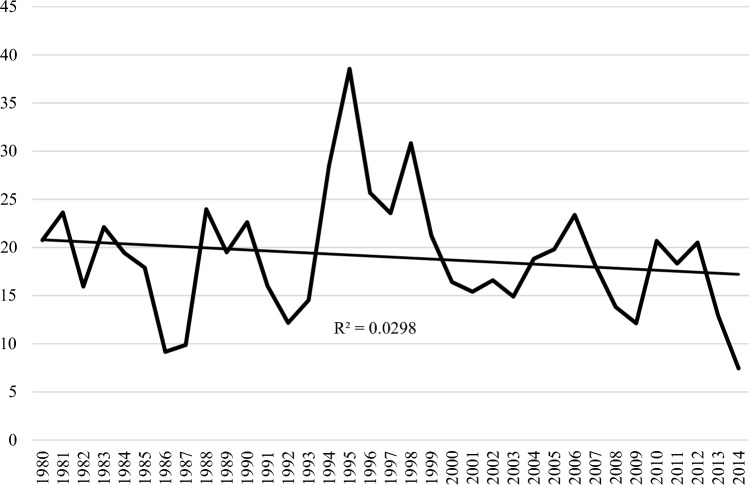
Annualised incidence rate per million population of immune-mediated inflammatory myopathies (IMM) in adults in Western Australia over the study period. Stippled line is trendlines with *R*^2^ and *p* value for trend

**Fig. 2:**
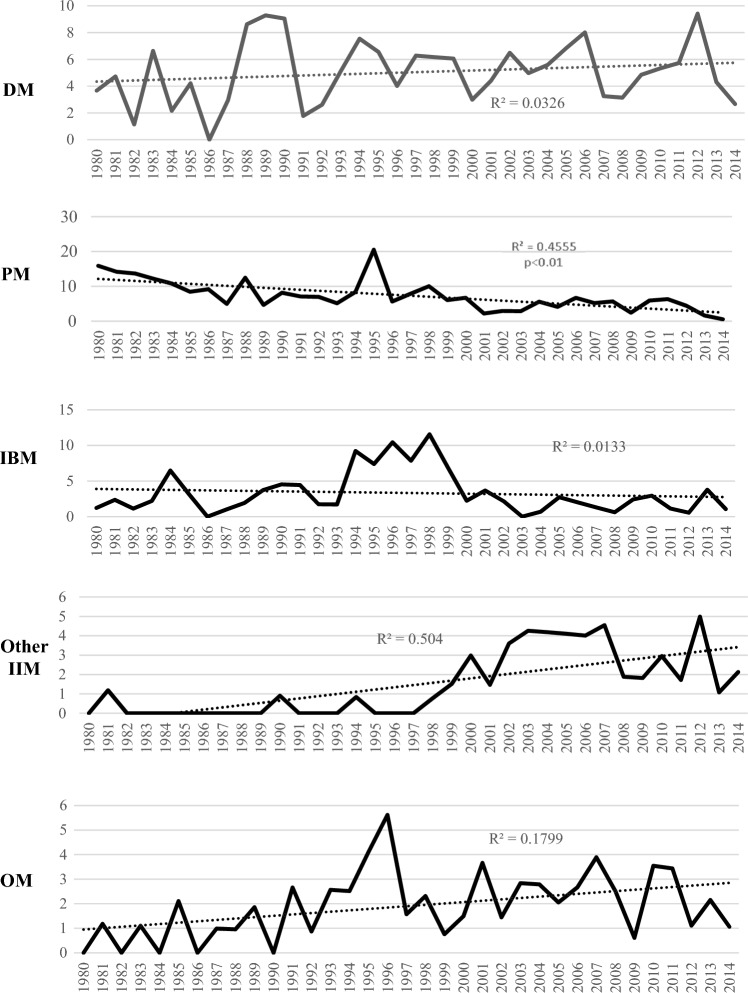
Annualised incidence rate per million of specific IIM subgroups including dermatomyositis (DM), polymyositis (PM), inclusion body myositis (IBM), other IIM and overlap myositis (OM) in adults in Western Australia over the study period. Stippled lines are trendlines with *R*^2^ and *p* values for trend

A total of 847 adults (a-IMM) received at least one discharge diagnosis of IIM 56.3% female, median age 64 (IQR 49–74, 3.5% Indigenous) (Table 1). This resulted in an averaged AIR for IIM of 19.0 (CI 10.4–27.5) which did not change significantly over the study period (Fig. 1). Patients were subclassified according to the initial diagnosis as having DM (*n* = 233, 27.5%), PM (*n* = 300, 35.5%), IBM (*n* = 146, 17.3%), other IIM (*n* = 77, 9.1%) or OM (*n* = 91,10.8%). The subgroup specific AIR was 5.0 (CI 0.6–9.4) for DM, 7.3 (CI 2.0–12.6) for PM, 3.3 (CI 0.7–9.6) for IBM, 1.5 (CI 0. 3–6.4) for other IIM and 1.9 for OM (CI 0.2–5.4). Over the study period, the AIR for PM rate declined and increased significantly for other IIM and OM (Fig. 2).

**Table 1:** Epidemiology, patient characteristics at time of index admission for IIM and outcomes in adult IIM patients

	All IIM (*n* = 847)	Controls (*n* = 3681)	DM (*n* = 233)	PM (*n* = 300)	IBM (*n* = 146)	Other IIM (*n* = 77)	OM (*n* = 91)
Annual incidence rate	19.0 (10.4–27.5)	–	5.0 (0.6–9.4)	7.3 (2.0–12.6)	3.3 (0.7–9.6)	1.5 (0.3–6.4)	1.9 (0.2–5.4)
Point prevalence rate	205.3 (185.6–226.6)	–	63.3 (52.7–75.5)	70.7 (59.4–83.5)	23.9 (17.7–31.8)	25 (18.6–33.0)	22.3 (16.3–29.9)
Demographics							
Female	477 (56.3)	1940 (52.8)	141 (60.5)*	169 (56.3)	68 (46.6)	34 (44.2)	65 (71.4)*
Age (yrs.)	64 (49–74)	65 (54–77)	60 (48–73)	63 (49–72.5)	68 (59–77)**	61 (51–78)	60 (40.5–71)
Indigenous	30 (3.5)	132 (3.6)	6 (2.6)	10 (3.3)	5 (3.4)	5 (6.5)	4 (4.4)
Medicare reliant	528 (64.9)	2267 (61.8)	143 (61.9)	179 (63)	98 (67)	54 (70.1)	64 (70.3)
Prior cancer	75 (8.9)	312 (8.5)	21 (9.0)	32 (10.7)	12 (8.2)	3 (3.9)	7 (7.7)
Outcome							
Initial stay (days)	7 (2–17)	2 (1–5)**	7 (2–18)	8 (3–16)	9 (2–17)	7 (3–15)	7 (3–18)
Follow-up (years)	5.75 (1.6–13.2)	4.6 (1.5–10.5)	5.0	8.42	5.17	6.33	5.17
Total p. years	6945	25,702	1774	3112	1010	470	579
Deaths	462 (54.5%)^#^	1519 (41.3%)	110 (47.2)	153 (51)	101 (72.1)	30 (39)	49 (53.8)
MR	6.65 (6.04–7.21)^#^	5.91 (5.62–6.33)	6.22 (5.19–7.49)	4.92 (4.25–5.83)	10.1 (8.38–12.14)	6.39 (4.32–9.65)	8.46 (6.32–10.21)

Figures indicate number (%), median (interquartile range), incidence and prevalence rates/million and mortality rate/100 p. years, all with 95% confidence intervals

Point prevalence per 1-1-2015

*Indicates significant difference versus PM, IBM and other IIM (*p* ≤ 0.01)

**Indicates significant difference with DM, PM and CTD-M in pairwise comparisons (*p* < 0.01)

^#^*p* < 0.05 versus controls

Patient characteristic at index diagnosis (Table 1) demonstrated a significantly higher proportion of females with DM and OM, while age at diagnosis was higher in IBM than in DM, PM and OM patients. Compared to controls, the proportion of IIM patients with a prior cancer diagnosis was not significantly increased (OR 1.05, CI 0.80–1.36) with a median timeframe between prior cancer and IIM diagnosis of 57 months (IQR 33–128). Also, the proportion of Indigenous patients (population parity 3.8%) and reliance on public health care (Medicare) were similar in both groups. An association with antilipemic medication was recorded for 8 (0.9%) of IIM patients overall with the highest proportion in DM (2.1%) and other IIM (1.3%). Although IIM patients required a longer initial hospital stay than controls, the length of stay did not differ between the various IIM subgroups.

**Fig. 3:**
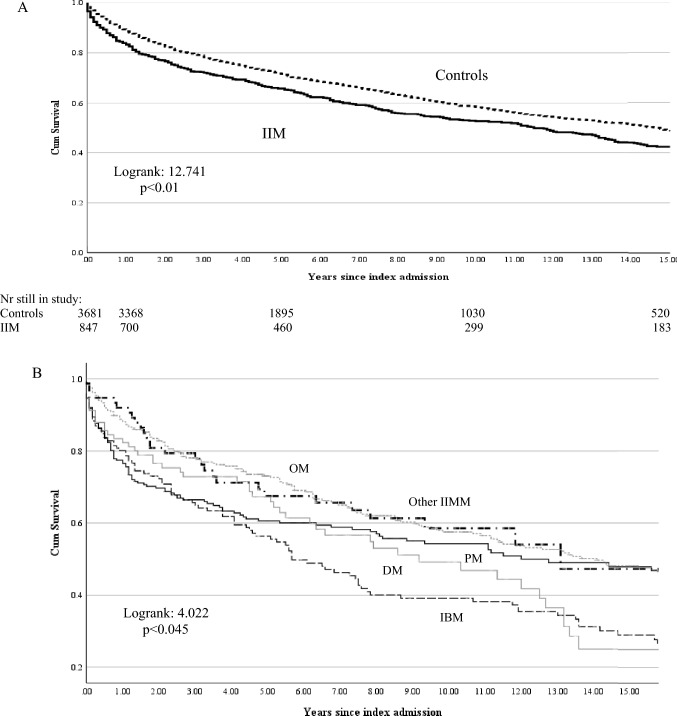
Kaplan–Meier survival curves for **A** IIM patients (straight line) and controls (broken line) and **B** for the IIM subgroups dermatomyositis (DM), polymyositis (PM), inclusion body myositis (IBM), and other IIM and overlap myositis (OM)

During 6945 person years, 452 deaths (54.5%) occurred in the IIM and 1519 (41.3%) in the control group (odds ratio for death 1.70, CI 1.47–1.98; *p* < 0.01) with a time adjusted MR of 6.65 (6.04–7.21) per 100 p.years in IIM patients versus 5.91 (5.62–6.33) in controls (Table 1) giving a MR ratio of 1.12 (CI 1.01–1.24; *p* = 0.03). Survival was lower at 5 years (65.8% vs 71.6%) and 10 years (52.5% vs 58.7%) post-diagnosis for IIM patients (Fig. 3A) with a HR for death of 1.21 (CI 1.09–1.35). Mortality rates were highest in subgroups of IBM and OM patients (Table 1) in line with poorest 5- and 10-year survival rates for IBM (56.4 and 39.2%; HR for death 1.82, CI 1.49–2.23) and OM patients (67 and 49.2%: HR for death 1.48, CI 1.11–1.97) compared to DM (60.6% and 54.2%, HR for death 1.17, CI 0.97–1.42), PM (73.2 and 57.5%, HR for death 0.98, CI 0.82–1.16), other IIM (67.7 and 58.6%, HR for death 1.10, CI 0.76–1.57) (Fig. 3B).

**Fig. 4:**
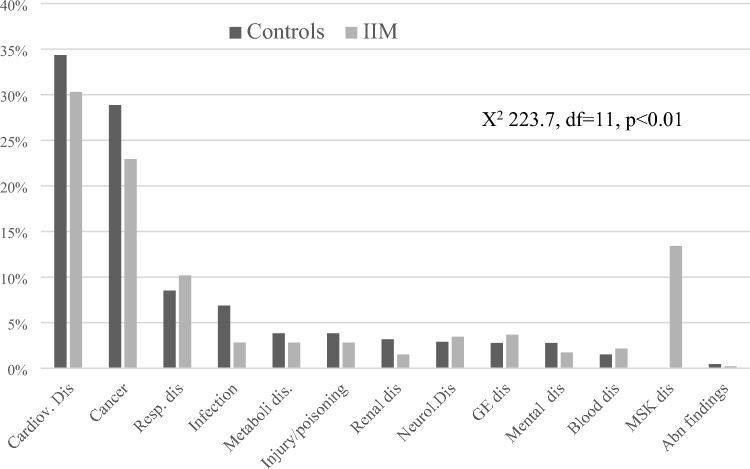
Primary causes of death as proportion of all deaths in IIM patients and controls

Primary causes of death were registered for 99.7% (1976/1981) of deaths. While cardiovascular disease and malignancy were the main causes of death in both groups, they were proportionally lower in IIM patients, while an increased proportion of deaths in IIM patients was observed for respiratory, gastrointestinal, and rheumatic diseases (Fig. 4).

## Discussion

This population-wide observational study found little variation in the overall incidence of IIM over 35 years, although the spectrum of IIM subgroups did change significantly with increases in other IIM and OM incidence and a corresponding decrease for PM. Mortality with IIM was higher with respiratory and rheumatic diseases more frequent causes of death than in matched controls. IIM point prevalence reached 205 per million in 2015.

Earlier studies from Australia studies using biopsy confirmed cases fulfilling Bohan and Peter criteria (BPC) for IIM [25] have reported an incidence of 7–8/million [9, 26]. A 2015 meta-analysis of sixteen studies found large incidence variations due to differences in case ascertainment, inclusion (biopsy only) and exclusion criteria (e.g. juvenile IIM) but estimated the overall AIR for adult IIM at 19.97 per million [27]. The lowest incidence rates were observed in hospital-based evaluations and the highest rates in administrative databases in the UK, USA and Taiwan. More recent population studies reported AIR of 11–15/million in Sweden depending on case definitions [28], while IIM incidence was 17.6/million in the UK [29]. These studies and the meta-analysis approximate the overall AIR of 19/million observed in this study, while the 2015 prevalence of adult IIM of 250/million also is in line with the meta-analysis estimate. Our study was based on clinical diagnosis of IIM translated into ICD codes by trained coders, which has been shown to have good validity for inpatient data [21]. Furthermore, in selecting IIM patients, we applied the ICD-based algorithm with the highest agreement with clinical diagnosis [24] and multiple studies have shown good, but not complete agreement between BPC, the more recent EULAR/ACR criteria and clinician diagnosis [30, 31]. Thus, we feel our data provided a valid approximation of IIM epidemiology in the general population.

IIM are heterogeneous in terms of clinical manifestations, and although newer more stringent classification criteria for subgroups were proposed in 2017 [31], the basic subclassification proposed by PBC [25] has largely remained unaltered. PM was the biggest subgroup (35.4%), followed by DM (27.5%) and IBM (17.2%) in this study. PM also was the larger subgroup in other studies from that period [26, 32], but our data indicate a decreasing incidence of PM over time and an increase in other IIM. This is likely because of the increasing recognition and spectrum of IMM associated autoantibodies and emergence of necrotising autoimmune myositis (NAM) and anti-synthetase syndrome (ASS) as separate entities [33]. As there has been no guidelines for ICD-10AM coding for NAM/ASS in Australia until 2021, most cases have been assigned to other myopathies which explains the rising incidence of this subgroup. The AIR for DM (5/million) was stable over time in agreement with data from a population based study in Norway [34] and a recent hospital based study from Olmsted county [35]. IBM incidence (3.3/million) was in line with earlier data from South Australia (2.9/million) [9] and Sweden (2.5million) [36], while prevalence (20.1/million) approximated the figures from two meta-analyses (20.1 and 24.8 /million) [12, 27] but was somewhat higher than reported earlier from WA (14.9/million) [7]. These data suggest that no clear change occurred in IIM epidemiology, and shifts in subgrouping have occurred as a result of increasing insight in disease processes [33].

The demographics of our study cohort were unremarkable with highest age and male preponderance observed in IBM and other IIM and more females in other subgroups as reported elsewhere [11, 12]. The overall proportion of Indigenous patients with IIM was at population parity, and although slightly higher in the other IIM and OM subgroups, this was not significant and does not suggest a potential genetic or environmental contribution to disease pathogenesis.

IIM are serious conditions that reduce quality of life, can affect multiple organ systems, and usually require immune modulating drug therapy that carries numerous side effects [3, 37–39]. While early studies based on hospital case series for IIM and/or subgroups reported increased mortality [40, 41], survival data across all the IIM subgroups in the general population are limited. We found that developing IIM increased the mortality rate by 12% compared to age and gender matched hospitalised comparators and that 10-year survival was just over 50% with worst 10-year survival of 39% seen in IBM patients (Fig. 3). A Norwegian population based study in 326 patients between 2003 and 2012 found increased standardised mortality rate (SMR) for DM (2.6) than PM (2.4) and IBM (1.7) and reported 5 year survival rates of 77% and 10 year of 60% while also noting the worst prognosis for IBM patients with a 40% 10 year survival rate [42]. A Swedish study based on 716 patients from a national IIM registry described a hazard ratio of death of 2.9 and a 10-year survival rate around 55% for IIM patients with worse prognosis for DM patients versus other IIM subgroups [43]. These combined data support the increased mortality for IIM patients in general and especially for IBM and DM patients [35, 44, 45].

Cardiovascular disease and cancer were numerically the predominant underlying causes of death in both IIM patients and controls, confirming the pattern observed in other studies [42, 43, 46]. An association between IIM and cancer risk has long been recognised, but the underlying mechanism and predilection for certain IIM subgroups remain unexplained [15]. Cardiovascular disease risk has been well described in IIM due to cardiac complications from the disease and required drug therapy [13, 47, 48]. Both areas potentially offer the greatest clinical opportunities to significantly reduce mortality in IIM patients by appropriate screening and intervention. Respiratory and rheumatic disease were overrepresented as causes of death in IIM patients, suggesting that pulmonary and musculoskeletal disease progression remain significant contributors to the worse prognosis. A recent study found that the rate at which PM/DM was a contributory cause of death doubled, when analysing multiple versus underlying cause of death [45].

The obvious limitations of this study include the lack of clinical details such as biochemical measures of disease activity, myositis specific and associated antibody profiles and biopsy results as potential classifying and prognostic markers. The nature of the dataset also precluded patient selection by classification criteria, but we included patients according to an algorithm with high predictive value and sensitivity in identifying UK patients with an IIM diagnosis by expert clinicians [24]. Our analyses used the initial diagnosis to classify IIM subgroups, and we did not consider the possibility that patients later were found to be misclassified or crossed over to other subgroups. The strength of this study lies in its ability to study longitudinal validated data on disease frequency and outcomes in a large group of patients suffering from a rare disease.

In conclusion, IIM affects 205 persons per million population in WA. While the overall incidence of IIM was stable over 35 years, we noticed significant changes in the incidence of subgrouping over time. Whether the observed changes will help improve the currently guarded prognosis for IIM patients will require further subgroup study with long-term follow-up.

### Supplementary Information

Below is the link to the electronic supplementary material.

Supplementary file1 (DOCX 22 KB)

## Data Availability

The data that support the findings of this study were used under license from WA Health Data Linkage Branch. Restrictions apply to the availability of these data, but upon reasonable request and following permission of WA Health and WA Data Linkage Branch data are available from the authors. Approval for use of de-identified data was obtained from the Human Research Ethics Committee at the WA Department of Health (WADOH HREC# 2016.24). As this study was considered low risk by the WA Health HREC and due to the de-identified nature of the linked health data set, the requirement for patient consent was waived. WA Health is proprietor of this administrative health data dataset. All named authors meet the International Committee of Medical Journal Editors (ICMJE) criteria for authorship for this article, take responsibility for the integrity of the work, and have given their approval for this version to be published.

## References

[1] Tieu J, Lundberg IE, Limaye V (2016). Idiopathic inflammatory myositis. Best Pract Res Clin Rheumatol.

[2] Day JA, Limaye V (2019). Immune-mediated necrotising myopathy: a critical review of current concepts. Semin Arthritis Rheum.

[3] Baig S, Paik JJ (2020). Inflammatory muscle disease—an update. Best Pract Res Clin Rheumatol.

[4] Miller FW, Lamb JA, Schmidt J, Nagaraju K (2018). Risk factors and disease mechanisms in myositis. Nat Rev Rheumatol.

[5] McLellan K, Papadopoulou C (2022). Update on biomarkers of vasculopathy in juvenile and adult myositis. Curr Rheumatol Rep.

[6] Miller FW (2021). Slicing and dicing myositis for cures and prevention. Nat Rev Rheumatol.

[7] Needham M, Corbett A, Day T, Christiansen F, Fabian V, Mastaglia FL (2008). Prevalence of sporadic inclusion body myositis and factors contributing to delayed diagnosis. J Clin Neurosci.

[8] Phillips BA, Zilko PJ, Mastaglia FL (2000). Prevalence of sporadic inclusion body myositis in Western Australia. Muscle Nerve.

[9] Tan JA, Roberts-Thomson PJ, Blumbergs P, Hakendorf P, Cox SR, Limaye V (2013). Incidence and prevalence of idiopathic inflammatory myopathies in South Australia: a 30-year epidemiologic study of histology-proven cases. Int J Rheum Dis.

[10] Buchbinder R, Forbes A, Hall S, Dennett X, Giles G (2001). Incidence of malignant disease in biopsy-proven inflammatory myopathy. A population-based cohort study. Ann Intern Med.

[11] Molberg O, Dobloug C (2016). Epidemiology of sporadic inclusion body myositis. Curr Opin Rheumatol.

[12] Callan A, Capkun G, Vasanthaprasad V, Freitas R, Needham M (2017). A systematic review and meta-analysis of prevalence studies of sporadic inclusion body myositis. J Neuromuscul Dis.

[13] Weng MY, Lai EC, Kao Yang YH (2019). Increased risk of coronary heart disease among patients with idiopathic inflammatory myositis: a nationwide population study in Taiwan. Rheumatology (Oxford).

[14] Naddaf E, Shelly S, Mandrekar J, Chamberlain AM, Hoffman EM, Ernste FC, Liewluck T (2022). Survival and associated comorbidities in inclusion body myositis. Rheumatology (Oxford).

[15] Moghadam-Kia S, Oddis CV, Ascherman DP, Aggarwal R (2020). Risk factors and cancer screening in myositis. Rheum Dis Clin North Am.

[16] Kelman CW, Bass AJ, Holman CD (2002). Research use of linked health data—a best practice protocol. Aust N Z J Public Health.

[17] Holman CD, Bass AJ, Rouse IL, Hobbs MS (1999). Population-based linkage of health records in Western Australia: development of a health services research linked database. Aust N Z J Public Health.

[18] Taylor-Williams O, Inderjeeth CA, Almutairi KB, Keen H, Preen DB, Nossent JC (2022). Total hip replacement in patients with rheumatoid arthritis: trends in incidence and complication rates over 35 years. Rheumatol Ther.

[19] Nossent J, Inderjeeth C, Keen H, Preen D, Li I, Kelty E (2022). The association between TNF inhibitor therapy availability and hospital admission rates for patients with ankylosing spondylitis. A longitudinal population-based study. Rheumatol Ther.

[20] Nossent J, Raymond W, Keen H, Preen DB, Inderjeeth CA (2021). Non-gonococcal septic arthritis of native joints in Western Australia. A longitudinal population-based study of frequency, risk factors and outcome. Int J Rheum Dis.

[21] Kwa MC, Ardalan K, Laumann AE, Nardone B, West DP, Silverberg JI (2017). Validation of international classification of diseases codes for the epidemiologic study of dermatomyositis. Arthritis Care Res (Hoboken).

[22] Hsu J-L, Liao M-F, Chu C-C, Kuo H-C, Lyu R-K, Chang H-S, Chen C-M, Wu Y-R, Chang K-H, Weng Y-C, Chang C-W, Chiang H-I, Cheng C-K, Lee P-W, Huang C-C, Ro L-S (2021). Reappraisal of the incidence, various types and risk factors of malignancies in patients with dermatomyositis and polymyositis in Taiwan. Sci Rep.

[23] Dobloug GC, Antal EA, Sveberg L, Garen T, Bitter H, Stjarne J, Grovle L, Gran JT, Molberg O (2015). High prevalence of inclusion body myositis in Norway; a population-based clinical epidemiology study. Eur J Neurol.

[24] Hannah JR, Gordon PA, Galloway J, Rutter M, Peach EJ, Rooney M, Stilwell P, Grainge MJ, Lanyon PC, Bythell M, Pearce FA (2022). Validation of methods to identify people with idiopathic inflammatory myopathies using hospital episode statistics. Rheumatol Adv Pract.

[25] Bohan A (1988). History and classification of polymyositis and dermatomyositis. Clin Dermatol.

[26] Patrick M, Buchbinder R, Jolley D, Dennett X, Buchanan R (1999). Incidence of inflammatory myopathies in Victoria, Australia, and evidence of spatial clustering. J Rheumatol.

[27] Meyer A, Meyer N, Schaeffer M, Gottenberg JE, Geny B, Sibilia J (2015). Incidence and prevalence of inflammatory myopathies: a systematic review. Rheumatology (Oxford).

[28] Svensson J, Arkema EV, Lundberg IE, Holmqvist M (2017). Incidence and prevalence of idiopathic inflammatory myopathies in Sweden: a nationwide population-based study. Rheumatology (Oxford).

[29] Parker MJS, Oldroyd A, Roberts ME, Ollier WE, New RP, Cooper RG, Chinoy H (2018). Increasing incidence of adult idiopathic inflammatory myopathies in the City of Salford, UK: a 10-year epidemiological study. Rheumatol Adv Pract.

[30] Parker MJS, Oldroyd A, Roberts ME, Lilleker JB, Betteridge ZE, McHugh NJ, Herrick AL, Cooper RG, Chinoy H (2019). The performance of the European League Against Rheumatism/American College of Rheumatology idiopathic inflammatory myopathies classification criteria in an expert-defined 10 year incident cohort. Rheumatology (Oxford).

[31] Lundberg IE, Tjärnlund A, Bottai M, Werth VP, Pilkington C, Visser M, Alfredsson L, Amato AA, Barohn RJ, Liang MH, Singh JA, Aggarwal R, Arnardottir S, Chinoy H, Cooper RG, Dankó K, Dimachkie MM, Feldman BM, Torre IG, Gordon P, Hayashi T, Katz JD, Kohsaka H, Lachenbruch PA, Lang BA, Li Y, Oddis CV, Olesinska M, Reed AM, Rutkowska-Sak L, Sanner H, Selva-O'Callaghan A, Song YW, Vencovsky J, Ytterberg SR, Miller FW, Rider LG (2017). 2017 European League Against Rheumatism/American College of Rheumatology classification criteria for adult and juvenile idiopathic inflammatory myopathies and their major subgroups. Ann Rheum Dis.

[32] Oddis CV, Conte CG, Steen VD, Medsger TA (1990). Incidence of polymyositis-dermatomyositis: a 20-year study of hospital diagnosed cases in Allegheny County, PA 1963–1982. J Rheumatol.

[33] Lundberg IE, Fujimoto M, Vencovsky J, Aggarwal R, Holmqvist M, Christopher-Stine L, Mammen AL, Miller FW (2021). Idiopathic inflammatory myopathies. Nat Rev Dis Primers.

[34] Dobloug C, Garen T, Bitter H, Stjarne J, Stenseth G, Grovle L, Sem M, Gran JT, Molberg O (2015). Prevalence and clinical characteristics of adult polymyositis and dermatomyositis; data from a large and unselected Norwegian cohort. Ann Rheum Dis.

[35] Kronzer VL, Kimbrough BA, Crowson CS, Davis JM, Holmqvist M, Ernste FC (2023). Incidence, prevalence, and mortality of dermatomyositis: a population-based cohort study. Arthritis Care Res (Hoboken).

[36] Lindgren U, Pullerits R, Lindberg C, Oldfors A (2022). Epidemiology, survival, and clinical characteristics of inclusion body myositis. Ann Neurol.

[37] Saygin D, Oddis CV (2022). Glucocorticoids in myositis: initiation, tapering, and discontinuation. Curr Rheumatol Rep.

[38] Pipitone N, Salvarani C (2020). Up-to-date treatment and management of myositis. Curr Opin Rheumatol.

[39] Yanagihara T, Inoue Y (2020). Insights into pathogenesis and clinical implications in myositis-associated interstitial lung diseases. Curr Opin Pulm Med.

[40] Yamasaki Y, Yamada H, Ohkubo M, Yamasaki M, Azuma K, Ogawa H, Mizushima M, Ozaki S (2011). Longterm survival and associated risk factors in patients with adult-onset idiopathic inflammatory myopathies and amyopathic dermatomyositis: experience in a single institute in Japan. J Rheumatol.

[41] Sultan SM, Ioannou Y, Moss K, Isenberg DA (2002). Outcome in patients with idiopathic inflammatory myositis: morbidity and mortality. Rheumatology (Oxford).

[42] Dobloug GC, Garen T, Brunborg C, Gran JT, Molberg Ø (2015). Survival and cancer risk in an unselected and complete Norwegian idiopathic inflammatory myopathy cohort. Semin Arthritis Rheum.

[43] Dobloug GC, Svensson J, Lundberg IE, Holmqvist M (2018). Mortality in idiopathic inflammatory myopathy: results from a Swedish nationwide population-based cohort study. Ann Rheum Dis.

[44] Price MA, Barghout V, Benveniste O, Christopher-Stine L, Corbett A, de Visser M, Hilton-Jones D, Kissel JT, Lloyd TE, Lundberg IE, Mastaglia F, Mozaffar T, Needham M, Schmidt J, Sivakumar K, DeMuro C, Tseng BS (2016). Mortality and causes of death in patients with sporadic inclusion body myositis: survey study based on the clinical experience of specialists in Australia, Europe and the USA. J Neuromuscul Dis.

[45] Qiao P, Guo Q, Gao J, Ma D, Liu S, Gao X, Lu TH, Zhang L (2023). Long-term secular trends in dermatomyositis and polymyositis mortality in the USA from 1981 to 2020 according to underlying and multiple cause of death mortality data. Arthritis Res Ther.

[46] Limaye V, Hakendorf P, Woodman RJ, Blumbergs P, Roberts-Thomson P (2012). Mortality and its predominant causes in a large cohort of patients with biopsy-determined inflammatory myositis. Intern Med J.

[47] Nuno L, Joven B, Carreira P, Maldonado V, Larena C, Llorente I, Tomero E, Barbadillo MC, Garcia-de la Pena P, Ruiz L, Lopez-Robledillo JC, Moruno H, Perez A, Cobo-Ibanez T, Almodovar R, Lojo L, Monteagudo I, Garcia-De Yebenes MJ, Lopez-Longo FJ (2017). Multicenter registry on inflammatory myositis from the Rheumatology Society in Madrid, Spain: descriptive analysis. Reumatol Clin.

[48] Leclair V, Svensson J, Lundberg IE, Holmqvist M (2019). Acute coronary syndrome in idiopathic inflammatory myopathies: a population-based study. J Rheumatol.

