# Supplementary material for: The spectrum of idiopathic inflammatory myopathies in Western Australia: epidemiological characteristics and mortality over time
Source: Rheumatol Int. 2023 Oct 11;44(2):329–37. doi: 10.1007/s00296-023-05475-3 (PMC10796655; doi:10.1007/s00296-023-05475-3)
Supplement: Supplementary file 1 — Supplementary file1 (DOCX 22 KB) [file 296_2023_5475_MOESM1_ESM.docx]

Suppl Table 1 ICD based diagnostic and procedure codes to define patients admitted with idiopathic inflammatory myopathy and subgroups.

| **Feature** | ICD 9CM/10AM diagnostic and procedure codes |
| --- | --- |
| Any IIM | Hannah et al 2023 algorithm (ref…) |
| Dermatomyositis | 710.3 / M33.0 , M33.1 , M33.9 , M36.0 |
| Polymyositis | 710.4 / M33.2 , M60.1 , M60.8, M60.9 (+ILD only) |
| Inclusion body myositis: | 359.7, 359.8/ G72.4 |
| Other IIM | 359.8 / M60.6,M60.9 |
|  |  |
| Overlap Myositis | 359.6 , G73.7 |
| ILD | 515,515.9,516.3,516.9,517.0,517.8, J84.1,J84.9, J99.1 |
